# Motor and nonmotor complications in Parkinson’s disease: an argument for continuous drug delivery?

**DOI:** 10.1007/s00702-013-0981-5

**Published:** 2013-03-02

**Authors:** K. Ray Chaudhuri, Alexandra Rizos, Kapil D. Sethi

**Affiliations:** 1 King’s College Hospital, 9th Floor Ruskin Wing, Denmark Hill, London SE5 9RS UK; 2 Department of Neurology, Georgia Regents University, 1429 Harper Street, HF-1121, Augusta, GA 30912 USA; 3 National Parkinson Foundation Centre of Excellence and National RLS, King’s College Hospital, 9th Floor Ruskin Wing, Denmark Hill, London, SE5 9RS UK

**Keywords:** Parkinson’s disease, Motor complications, Nonmotor fluctuations, Continuous drug delivery

## Abstract

The complications of long-term levodopa therapy for Parkinson’s disease (PD) include motor fluctuations, dyskinesias, and also nonmotor fluctuations—at least equally common, but less well appreciated—in autonomic, cognitive/psychiatric, and sensory symptoms. In seeking the pathophysiologic mechanisms, the leading hypothesis is that in the parkinsonian brain, intermittent, nonphysiological stimulation of striatal dopamine receptors destabilizes an already unstable system. Accordingly, a major goal of PD treatment in recent years has been the attainment of continuous dopaminergic stimulation (CDS)—or, less theoretically (and more clinically verifiable), continuous drug delivery (CDD). Improvements in the steadiness of the plasma profiles of various dopaminergic therapies may be a signal of progress. However, improvements in plasma profile do not necessarily translate into CDS, or even into CDD to the brain. Still, it is reassuring that clinical studies of approaches to CDD have generally been positive. Head-to-head comparative trials have often failed to uncover evidence favoring such approaches over an intermittent therapy. Nevertheless, the findings among recipients of subcutaneous apomorphine infusion or intrajejunal levodopa/carbidopa intestinal gel suggest that nonmotor PD symptoms or complications may improve in tandem with motor improvement. In vivo receptor binding studies may help to determine the degree of CDS that a dopaminergic therapy can confer. This may be a necessary first step toward establishing whether CDS is, in fact, an important determinant of clinical efficacy. Certainly, the complexities of optimal PD management, and the rationale for an underlying strategy such as CDS or CDD, have not yet been thoroughly elucidated.

## Introduction

In patients with Parkinson’s disease (PD), both motor and nonmotor complications are common, burdensome sequelae of long-term levodopa treatment. The motor complications are well recognized. In broad terms, they consist of motor fluctuations and dyskinesias, although within each of these categories varied patterns have been described (Olanow et al. 2009). By some estimates, more than 50 % of PD patients report one or another such problem after 5 years of levodopa use; and after 10 years, up to 80 % of patients report them (Obeso et al. 2000). Their impact can be substantial, reducing the patient’s mobility and the ability to perform activities of daily living (ADL). In addition, emotional well-being and health-related quality of life (HRQoL) can be severely impaired (Chapuis et al. 2005; Damiano et al. 2000).

The nonmotor complications are fluctuations resulting in symptoms such as mood disturbance, cognitive dysfunction, dysautonomia, and pain (Witjas et al. 2002). Considered together (Chaudhuri et al. 2011), they may be at least as common as motor complications (Gunal et al. 2002), but they appear to be under-reported (Chaudhuri et al. 2010). In a survey of PD patients with motor fluctuations (Witjas et al. 2002), 100 % of participants reported nonmotor fluctuations, which 28 % rated as being more disabling than the motor problems. Anxiety, excessive sweating, slowed cognition, fatigue, and akathisia were the most frequent nonmotor fluctuations reported. The burdens imposed by such fluctuations are hard to differentiate from those imposed by PD itself. Still, in a recent large-scale study of the overall nonmotor burden (Martinez-Martin et al. 2011), total score on the nonmotor symptoms scale (NMSS) (Martinez-Martin et al. 2009) showed a high correlation with HRQoL (*r* = 0.70), as measured by the 39-item PD questionnaire (PDQ-39) (Jenkinson et al. 1997a). Remarkably, the correlation exceeded that for motor dysfunction (*r* = 0.58), as measured by scales for outcomes in PD (SCOPA) motor score (Martínez-Martín et al. 2005).

Numerous studies have investigated the predictors and potential pathophysiologic mechanisms of levodopa complications in PD patients, in hope of developing clinical strategies to avoid them. For motor complications, especially dyskinesia, the reported risk factors include higher dosage and longer duration of levodopa treatment, longer duration and severity of a patient’s PD, and younger age at PD onset (Grandas et al. 1999). Although nonmotor PD manifestations may be related to nondopaminergic brain changes concomitant with or secondary to the striatal dopaminergic deafferentation considered to be a PD hallmark (Simuni and Sethi 2008), at least some nonmotor fluctuations (e.g., in autonomic function) have likewise been associated with higher dosage and longer duration of levodopa treatment and with younger age at PD onset (Chaudhuri and Schapira 2009; Gunal et al. 2002). In a survey of patients with motor fluctuations, all 50 subjects reported nonmotor fluctuations, most of which occurred in the patients’ “OFF” state, thereby exhibiting correlation with their motor dysfunction (and with time spans of levodopa inefficacy) (Witjas et al. 2002). However, nonmotor fluctuations of all types (e.g., autonomic, cognitive/psychiatric, or sensory) are also seen during “ON” time (Gunal et al. 2002).

Extensive preclinical research, e.g., in primate PD models (Jenner 2009), has been advancing the hypothesis that the motor complications of chronic levodopa therapy may derive from the therapy’s intermittent, nonphysiological stimulation of the parkinsonian brain’s striatal dopamine receptors (Chase et al. 1989; Grace 2008; Jenner 2008; Nutt et al. 2000; Olanow et al. 2006). On this basis, a major goal of PD treatment in recent years has been the attainment of continuous dopaminergic stimulation (CDS)—or, less theoretically (and more clinically verifiable), continuous drug delivery (CDD). Pharmacologically, CDD has been attempted by strategies including a variety of levodopa formulations and delivery methods, as well as by dopamine agonists with differing pharmacokinetic properties and receptor-affinity profiles. This review will survey the key therapeutic advances.

## Defining the complications

Among the complications of chronic levodopa therapy in PD, motor fluctuations (Olanow et al. 2009) are defined as alterations between periods of clinical response of motor symptoms to levodopa, during which the patient’s mobility and motor function are relatively good (i.e., during “ON” time), and periods when motor response deteriorates (“OFF” time). In early PD, such oscillations are not expected to occur. At this stage of the disease, the motor response to a single levodopa dose is typically long-lasting (>4 h), despite the drug’s plasma half-life of only 60–90 min, yielding a span of “ON” time sufficient to bridge the interval between successive doses throughout the patient’s waking day. Indeed, response is often stable even if one or more doses are missed (“long-duration response”). With advancing PD, however, the duration of motor-symptom control conferred by a levodopa dose progressively shortens toward an approximate matching of the drug’s plasma half-life (“short-duration response”). Patients may then begin to experience a predictable “wearing-off” effect. Patients may also have rapid, unpredictable fluctuations between “ON” and “OFF” states (“ON–OFF phenomenon”) (Marsden and Parkes 1976), and some doses may take longer to become effective (“delayed-ON”) or may not be effective at all (“no-ON”) (Obeso et al. 2008). Although it has been hypothesized that motor fluctuations might arise in advanced PD because loss of dopaminergic presynaptic terminals may decrease striatal capacity to store dopamine, “wearing-off” has also been seen in PD treated with apomorphine and other dopamine agonists not stored in dopaminergic terminals (Bravi et al. 1994). The implication is that the pathophysiology of motor fluctuations must have postsynaptic facets (Verhagen Metman et al. 1997).

Dyskinesias in PD patients reliant on chronic levodopa (Olanow et al. 2009) are involuntary movements most typically coinciding with maximum levodopa plasma level and maximum motor-symptom response (“peak-dose dyskinesia”). In such patients, the dyskinesia is typically choreiform, involving any part of the body, but may be dystonic or myoclonic. Dyskinesia can also occur when an “ON” state begins and again as it ends (“diphasic dyskinesia”). In such patients, the abnormal movements tend to be rhythmic, stereotypic, and asymmetric, and to affect the legs. In either case, the dyskinesia has long been viewed as the expression of a disruption of the normal ability of the basal ganglia to select and execute motor programs (Marsden 1982).

With increasing appreciation of the complexity of the dopaminergic influence on basal ganglia function, there has come a more specific suspicion that dyskinesia may be the eventual outcome of a fundamental inadequacy of standard levodopa as an exogenous replacement for endogenous dopamine, namely the treatment’s abnormally pulsatile stimulation of striatal dopamine receptors (Jenner 2008; Olanow and Obeso 2000). The physiologic stimulation of these receptors appears to be a tonic process with phasic fine-tuning (Goto et al. 2007; Schultz 1994), requiring that the striatum maintain a steady baseline supply of endogenous dopamine (Venton et al. 2003). Hence, the institution of abnormally pulsatile stimulation is thought to lead to dysregulation of genes and proteins in striatal neurons, in turn, producing an enduring alteration of firing patterns in basal ganglia output neurons (Jenner 2008; Olanow and Obeso 2000). In brief, the intermittency of standard levodopa therapy does not achieve the desired normalization of the parkinsonian basal ganglia but instead destabilizes the already unstable system (Olanow et al. 2009). Indeed, in PD models such as the primate models induced by the dopamine-neuron-specific neurotoxin 1-methyl-4-phenyl-1,2,3,6-tetrahydropyridine (MPTP), intermittent administration of short-acting dopaminergic agents (e.g., levodopa) has been found notably apt to induce dyskinesia (Pearce et al. 1998); yet even the short-acting agents have been found not to do so if they are given continuously (Bibbiani et al. 2005).

The nonmotor fluctuations seen during long-term levodopa usage in PD have been categorized as dysautonomic, cognitive/psychiatric, and sensory (Witjas et al. 2007). Although their causes remain obscure, dopaminergic dysfunction would appear to be involved, acting either directly or through the unbalancing effects of dopaminergic dysfunction on other neurotransmitter systems (Witjas et al. 2007). As a possible example of a direct link, neuroimaging data have identified dopaminergic dysfunction (reduced receptor availability) in the hypothalamus of the parkinsonian brain (Politis et al. 2008), an abnormality conceivably influencing dysautonomia. As a possible example of an indirect link, neuroanatomic and neurophysiologic data have combined in suggesting that in the normal brain, dopaminergic and serotonergic systems interact via reciprocal connections between the substantia nigra (and ventral tegmental area) and the brainstem raphé nuclei (Di Giovanni et al. 2008), conceivably affecting sleep homeostasis. For either direct or indirect linkage of dopaminergic dysfunction to nonmotor fluctuations, the fluctuations might be responsive to CDD. However, it may be instructive that in advanced PD, deep brain stimulation of the subthalamic nucleus appears to best alleviate nonmotor fluctuations affecting sensory, autonomic, and cognitive function, while neuropsychiatric fluctuations respond less consistently (Witjas et al. 2007).

## The evolution of CDD strategies

Because of a number of pharmacokinetic factors, intermittent oral dosing of levodopa does not provide stable plasma drug levels. The most significant problems are the short half-life of levodopa (~60 min) (Deleu et al. 2002) and the pulsatile and unpredictable absorption of levodopa from the small intestine due to erratic gastric emptying (Kurlan et al. 1988; Nyholm et al. 2003). As efforts to improve the agent’s solubility so as to enhance its absorption, levodopa has been studied as an orally administered liquid or methyl ester (Antonini et al. 2010a). Brain levodopa levels also vary owing to competition of levodopa with amino acids for transport across the intestinal wall and the blood–brain barrier (Frankel et al. 1989), but these perturbations are relatively minor and in most patients appear not to have a clinically relevant impact (Nutt et al. 1989).

Efforts to achieve a steady level of levodopa, or of dopaminergic PD therapies in general, began almost at the inception of the treatments themselves (Tolosa et al. 1998). Inhibition of levodopa metabolism was first attempted in the 1960s, using the peripheral dopa decarboxylase inhibitor (DDI) benserazide or subsequently, carbidopa. Then, in the early 1970s, a sustained-release oral formulation of levodopa was developed. Dopamine-receptor agonist therapies were tested as early as 1951 but did not become an option in routine PD treatment until the 1970s, when an oral formulation of bromocriptine was introduced. Continuous infusion of a dopaminergic therapy was first attempted in the mid 1970s, using intravenous delivery. All of these broad strategies continue to be developed.

### Oral levodopa in combination with metabolic inhibitors

Dopamine does not cross the blood–brain barrier. By contrast, levodopa does cross the barrier, provided that it has not been converted to dopamine in the periphery. Within the central nervous system (CNS), dopaminergic neurons can perform this conversion. In view of these circumstances, the CNS bioavailability of levodopa, and of dopamine as its desired CNS metabolite, can be enhanced by a number of strategies (Deleu et al. 2002): (1) blocking the peripheral conversion of levodopa into dopamine or another metabolite, (2) blocking the central metabolism of levodopa along pathways that do not produce dopamine, and (3) blocking the central metabolism of dopamine.

Dopa decarboxylase inhibitors, such as carbidopa or benserazide, are given to implement the first of these strategies, i.e., they block the peripheral conversion of levodopa into dopamine, allowing a larger influx of levodopa into the brain. By doing so, they permit reduction of total daily levodopa dosage (Cedarbaum 1987). They also reduce levodopa side effects arising from peripheral dopamine-receptor stimulation (e.g., nausea and vomiting) (Kaakkola et al. 1985; Markham et al. 1974). Pharmacokinetically, they increase the peak plasma levodopa concentration (*C*_max_), increase overall levodopa exposure [area under the concentration–time curve (AUC)], and prolong levodopa plasma half-life (Cedarbaum 1987; Robertson et al. 1989). In clinical practice, levodopa is invariably combined with carbidopa or benserazide, regardless of other strategies that may also be employed to enhance levodopa availability.

**Fig. 1:**
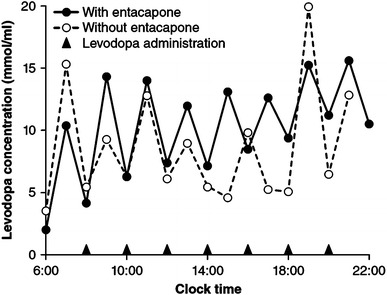
Plasma levels of immediate-release oral levodopa with versus without entacapone in a PD patient receiving treatment (*arrowheads*) every 2 h (data adapted from Nutt 1996)

Catechol-*O*-methyltransferase (COMT) inhibitors implement the first and perhaps also the second and third strategy, depending on whether the drug can enter the brain. Via COMT inhibition, each such drug blocks the conversion of levodopa into 3-*O*-methyldopa or 4-*O*-methyldopa and also of dopamine to 3-methoxytyramine (Deleu et al. 2002). However, entacapone acts only in the periphery, while tolcapone may have some central effect. On the other hand, tolcapone is associated with liver toxicity and is recommended only for motor complications not responsive to other levodopa adjuncts (and even then only with regular monitoring of liver function) (Tasmar^®^ 2009). Pharmacokinetically, single-dose entacapone has been found to increase the AUC and prolong the plasma half-life of levodopa administered as an immediate-release (IR) formulation, without affecting levodopa *C*_max_ (Nutt 1996, 2000). With repeated entacapone dosing, both peaks and troughs in levodopa plasma level become higher, with diminished difference between them (Fig. 1) (Nutt 1996). This leveling, however, is not as pronounced as may be achieved by levodopa administered as a controlled-release (CR) formulation (LeWitt et al. 2009). Pharmacokinetic-pharmacodynamic studies (Merello et al. 1994; Piccini et al. 2000; Ruottinen and Rinne 1996a, b) have shown that an increase in the AUC of levodopa administered with entacapone is temporally associated with improved motor function. This benefit, however, may be achieved at a cost of increased dyskinesia (Stocchi et al. 2010).

Monoamine oxidase (MAO) inhibitors implement the third strategy: i.e., they block the conversion of CNS dopamine into 3,4-dihydroxyphenylacetic acid (Deleu et al. 2002). Of the two known MAO isoforms, MAO-A predominates in the intestinal tract while MAO-B is prominent in brain areas including the basal ganglia (Youdim and Bakhle 2006). Two drugs, rasagiline and selegiline (Azilect^®^ 2009; Eldepryl^®^ 2011), are currently marketed as irreversible MAO-B-selective inhibitors. Both are approved as adjunctive therapy to levodopa, and rasagiline is also approved as monotherapy in early PD. In general, MAO-B inhibitors improve “ON” time only modestly (Rascol et al. 2005), suggesting that patients may continue to experience motor and nonmotor fluctuations.

### Controlled-release levodopa

**Fig. 2:**
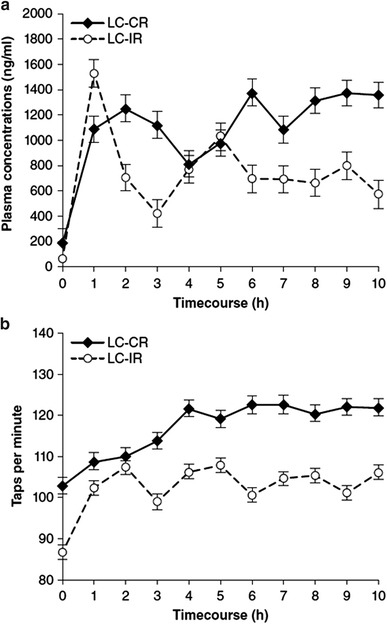
Plasma levels (**a**) and motor-function (tapping total) profiles (**b**) for slow- versus immediate-release oral levodopa/carbidopa in 18 PD patients with motor fluctuations (data adapted from Pahwa et al. 1997). *LC*-*CR* (slow) controlled-release levodopa/carbidopa, *LC*-*IR* immediate-release levodopa/carbidopa

Slow-release formulations of orally administered levodopa are available only in combination with a DDI (carbidopa in the United States and benserazide or carbidopa in the European Union). Madopar^®^ HBS (hydrodynamically balanced system; Roche Products, Ltd, Hertfordshire, UK) combines levodopa and benserazide in a capsule that forms a mucoid body, which in turn remains in the stomach for a prolonged period, allowing slow diffusion of its contents (Erni and Held 1987). Sinemet^®^ CR (Merck & Co., Inc., Whitehouse Station, NJ; Bristol-Myers Squibb Company, Princeton, NJ) combines levodopa and carbidopa in a monolithic-matrix tablet that releases the drugs gastrointestinally, via surface dissolution and erosion (Dempski et al. 1989; Wilding et al. 1991). In general, slow-release formulations may permit a decrease in dosing frequency and may reduce the temporal variability in levodopa plasma level (Fig. 2a), compared with levodopa in its standard IR form (Cedarbaum et al. 1989; Pahwa et al. 1997). Moreover, pharmacodynamic studies in advanced PD have reported clinical benefits (Fig. 2b), including an increase in “ON” time (Cedarbaum et al. 1989; Pahwa et al. 1997). However, slow-release formulations also delay the levodopa plasma *C*_max_ (~2 h vs. 30–45 min for IR formulations) and may exhibit a lower level at the maximum, necessitating an increase in total daily levodopa dosage (Pahwa et al. 1997; Sage and Mark 1994). A major problem is that erratic pharmacokinetics may result in unpredictable clinical response. Moreover, patients on slow-release formulations often require a morning dose of an IR formulation. A new oral levodopa–carbidopa formulation combining the pharmacokinetics of IR and CR formulations has shown promise in clinical trials (Hauser 2012) and awaits FDA approval.

### Dopamine-receptor agonists

Dopamine-receptor agonists are indicated as monotherapy in early PD and as adjunctive therapy to levodopa at all stages of PD. Despite the gold-standard status of levodopa for controlling motor symptoms, the agonists have potential advantages, including the pharmacokinetic advantage of their longer half-lives (Kvernmo et al. 2006). In addition, dopamine-receptor agonists exhibit selectivity in their binding to dopamine receptors, conceivably permitting a reduction in the expression of dyskinesias, e.g., by use of agonists selective for D_2_-like receptors (Jenner 2008). Several first-generation agonists (e.g., pergolide, cabergoline, and bromocriptine) are ergotamine derivatives associated with fibrotic heart disease (Zanettini et al. 2007) perhaps related to 5-HT(2B) serotonin-receptor agonism (Antonini and Poewe 2007). In consequence, pergolide has been withdrawn from the US market; and in the European Union, the first-generation agents are now restricted to second-line use (of pergolide and cabergoline) or now carry warning labels. Despite being ergoline, lisuride lacks 5-HT(2B) agonism and is not known to cause cardiac valvular fibrosis (Hofmann et al. 2006). Lisuride has been formulated for transdermal delivery from a skin patch (which, however, is not widely used, owing to neuropsychiatric complications).

**Fig. 3:**
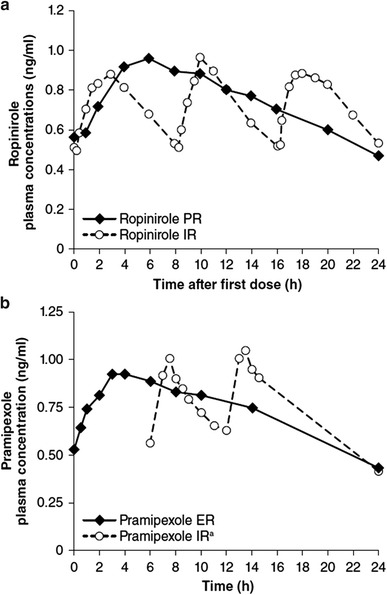
Plasma levels for PR (**a**)/ER (**b**) versus IR formulations of oral dopamine agonists: Ropinirole (**a**) in 20 patients with early PD (data adapted from Tompson and Vearer 2007), and pramipexole (**b**) in 14 healthy volunteers (data adapted from Jenner et al. 2009). *ER* extended-release, *IR* immediate-release, *PR* prolonged-release. ^a^Only the last two doses are graphed for pramipexole IR

Ropinirole and pramipexole are second-generation, nonergoline, D_2_-like-receptor preferring agonists widely prescribed for oral administration in PD. In their IR forms, the half-life of ropinirole is ~6 h and that of pramipexole is 8–12 h (Kvernmo et al. 2006). Both drugs are also available as slow-release formulations. Prolonged-release (PR) ropinirole is a tablet based on matrix technology. Compared with the IR formulation given three times daily (Tompson and Vearer 2007), its once-daily dosing provided a similar dose-normalized AUC_0–24 h_, a *C*_max_ 12 % lower, and a median *t*_max_ of approximately 6 versus 2 h for the IR formulation (Fig. 3a). In a double-blind study in early PD (Stocchi et al. 2008), it exhibited noninferiority to ropinirole IR, as judged by unified Parkinson’s disease rating scale (UPDRS) (Fahn et al. 1987) motor score. Extended-release (ER) pramipexole is also a matrix tablet. At steady-state for once-daily dosing (Jenner et al. 2009b), its *C*_max_ and AUC_0–24 h_ resembled those of the IR formulation given three times daily, but the geometric mean *t*_max_ was more than 5 h versus approximately 1 h for the IR formulation (Fig. 3b). In a double-blind study in early PD (Hauser et al. 2010), improvements in UPDRS motor plus ADL scores resembled those for the IR formulation.

Rotigotine is a nonergoline dopamine agonist selective for D_2_-like receptors, but also showing affinity for D_1_-like receptors (Scheller et al. 2009). Formulated for transdermal delivery from skin patches each to be worn for 1 day (Cawello et al. 2007, 2009; Pfieffer 2005), its availability has been impeded by problems with crystallization. Nevertheless, a recent large-scale study (Trenkwalder et al. 2011a) in patients selected for having unsatisfactory early morning motor-symptom control at any PD stage, with or without levodopa, constitutes the first double-blind, placebo-controlled investigation of the effects of a CDD strategy on both motor and nonmotor deficits. At 12 weeks, mean early morning motor dysfunction, mean sleep disturbance, and depressive symptomatology [as measured by UPDRS (Fahn et al. 1987), the PD sleep scale (PDSS) (Trenkwalder et al. 2011b), and the Beck depression inventory (BDI) (Visser et al. 2006), respectively] showed significantly greater improvements in the active-treatment group. Rotigotine has been re-introduced in the US market as a new formulation that may be more stable than the original.

Impulse control disorders such as compulsive gambling, shopping, or hypersexuality are being increasingly recognized in PD patients as adverse effects of dopamine agonist therapy (Weintraub and Nirenberg 2013). The degree of risk associated with long-acting dopamine agonists or infusional dopaminergic therapies is currently under investigation. A recent post-marketing survey conducted in Europe has suggested that the risk may be lower for the rotigotine patch and for pramipexole ER than for shorter-acting agonists (Rizos et al. 2012).

### Continuous drug infusion

Apomorphine and levodopa/carbidopa intestinal gel (LCIG) are the two dopaminergic therapies currently available as a continuous infusion for patients with severe motor fluctuations in advanced PD. Typically, each is administered during waking hours only. (A third therapy, lisuride infusion, is not widely used.)

**Fig. 4:**
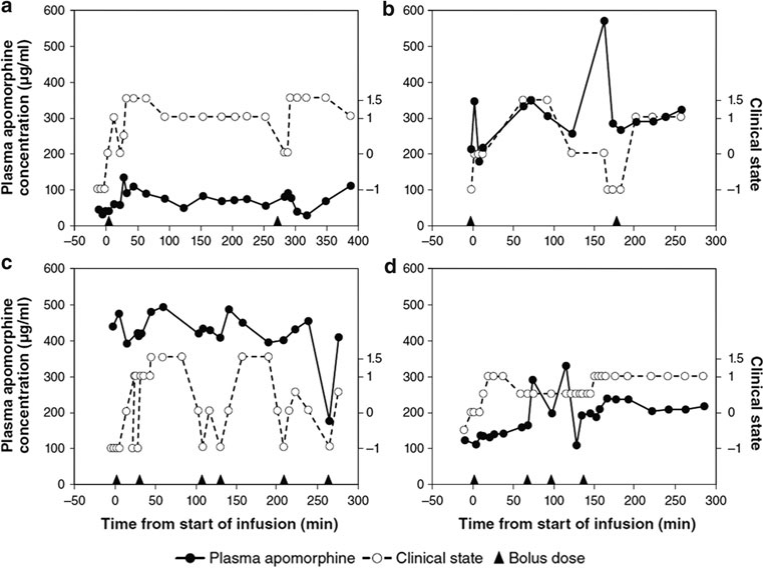
Plasma apomorphine levels (*left y-axis scale*) and simultaneous clinical-state scores (*right y-axis scale*) for each of four recipients of continuous subcutaneous apomorphine infusion. Arrowheads on *x-axis* mark bolus doses. Clinical state was a global objective/subjective rating in half-point increments, including ratings of −1 for fully “OFF”, 0 for threshold of “ON”, +1 for fully “ON”, and +1.5 for dyskinesia or subjectively overdosed (e.g., light-headed, confused) (data adapted from Manson et al. 2001)

Apomorphine (Apokyn^®^ (2012) is a nonergoline dopamine-receptor agonist with less receptor selectivity than that of other available agonists, although it does demonstrate some D_2_-receptor preference (Deleu et al. 2004). After subcutaneous injection, it is notably short-acting: in one study, its elimination half-life was 33 min (Gancher et al. 1989). A portable pump permits its continuous infusion into subcutaneous fatty tissue of the abdomen, thighs, or arms (LeWitt 2004); a delivery method available in Europe. After long-term usage, however, inflammatory skin nodules may form and may interfere with drug absorption (Nicolle et al. 1993). In a small study of PD patients switched from subcutaneous to intravenous apomorphine (delivered by indwelling venous catheter) for refractory motor fluctuations (Manson et al. 2001), dyskinesia showed substantial decrease, and “OFF” time was reported to be virtually eliminated (a mean reduction from 5.4 to 0.5 h; *p* < 0.05), although plasma apomorphine levels did not correlate well with dosage level or with motor function (Fig. 4a–d), and complication rates were high. In another small study, of two PD patients, plasma apomorphine levels likewise showed weak correlation with motor function, but for cerebrospinal-fluid apomorphine levels, the correlation with motor function was strong (Hofstee et al. 1994).

**Fig. 5:**
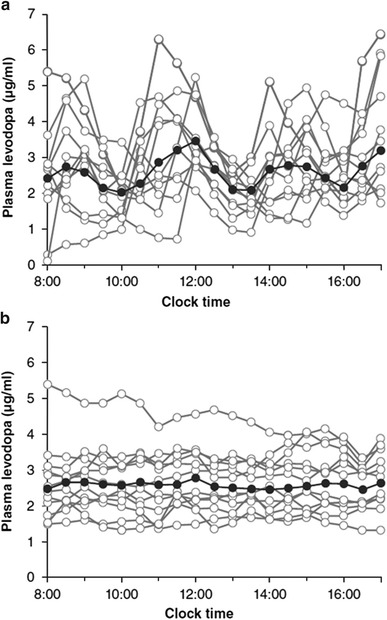
Plasma levodopa levels for sustained- (slow-) release oral levodopa/carbidopa (**a**) versus continuous intraduodenal infusion of LCIG (**b**) in each of 12 patients with advanced PD. In each graph, the *solid black curve* with *solid black circles* for its data points displays the mean for all curves (data adapted from Nyholm et al. 2003)

Levodopa/carbidopa intestinal gel (Duodopa^®^; Abbott, Abbott Park, IL) is a methylcellulose gel suspension of levodopa/carbidopa formulated for continuous enteral infusion from a portable pump and medication cassette worn attached to the waist or over the shoulder (Nyholm et al. 2003). To complete the delivery system, jejunal or duodenal tubing is emplaced by a percutaneous endoscopic gastrostomy procedure. In a small crossover trial (Nyholm et al. 2003) comparing nasoduodenal LCIG infusion with optimized sustained-release oral levodopa/carbidopa, the mean steady-state plasma levodopa concentration was the same for both the treatments, but the mean *C*_max_ was lower for LCIG (Fig. 5a, b); and on motor tasks performed at hourly intervals, a higher proportion of observations were considered near-normal [at 80 % for LCIG versus 61 % levodopa/carbidopa CR; estimated mean difference, 19%; 95 % confidence interval (CI): 12, 26 %; *p* < 0.01]. LCIG is currently available in Europe, Canada, and Australia and is under investigation in the United States.

## Clinical correlates of CDD

For dopaminergic therapies formulated and/or delivered in efforts to approach or attain CDD, numerous studies have evaluated the impact on motor fluctuations and dyskinesia. Both acute effects and the ability to delay the onset of such complications have been assessed. The impact on nonmotor function has not yet been evaluated extensively.

### Prevention of motor complications in early PD

The most recent practice guidelines for treating early PD are those of a consensus document published jointly in 2006 by the European Federation of Neurological Societies (EFNS) and the Movement Disorder Society (MDS) European Section (Horstink et al. 2006). Based on available evidence, this panel judged ropinirole and pramipexole, in their IR formulations, to be effective as monotherapy both for motor-symptom control and for prevention of levodopa-associated motor complications, especially among younger patients, in whom such complications are thought to be more likely. For other dopaminergic therapies, data either were lacking, as in the case of COMT inhibitors and the MAO-B inhibitor rasagiline, or did not support efficacy for motor-complication prevention, as in the case of levodopa CR and the MAO-B inhibitor selegiline.

Since then, long-term (6-year) data from the CALM-PD study of levodopa versus pramipexole IR as initial PD pharmacotherapy have found dopaminergic motor complications (encompassing “wearing-off”, “ON–OFF” effects, or dyskinesias) to be more likely for levodopa than for the dopamine agonist (Parkinson Study Group CALM Cohort Investigators 2009). However, disabling dyskinesias were uncommon in both treatment groups. Long-term (6.5-year) data from an open-label extension (Hauser et al. 2009a) of the TEMPO study (Parkinson Study Group 2004) of early- versus delayed-start rasagiline have failed to demonstrate a difference in the median time to development of motor complications (or time to addition of levodopa) in patients who started receiving rasagiline 6 months earlier in their PD than did the delayed-start group. Data have also been reported concerning the efficacy of COMT inhibitors in early PD. In the 39-week, double-blind FIRST-STEP study (Hauser et al. 2009b), the incidence of “wearing-off” and dyskinesia did not differentiate between groups receiving levodopa/carbidopa/entacapone (LCE) or only levodopa/carbidopa three times daily, despite the superiority of LCE on efficacy measures including the study’s primary outcome, the sum of motor and ADL scores in the UPDRS (but not on measures including the motor score alone). STRIDE-PD (Stocchi et al. 2010), a large-scale, double-blind study designed specifically to evaluate the capacity of LCE to delay dyskinesia, has also failed to find such benefit. For treatment lasting up to 208 weeks, the risk of dyskinesia was actually higher in the study’s LCE group than in its levodopa/carbidopa group. Although the mean dosage of levodopa was highly similar across these groups, the estimated bioavailability of levodopa was significantly heightened by the entacapone in LCE, conceivably hastening dyskinesia (*p* < 0.001). The investigators also hypothesized that the study’s four-times-daily dosing of LCE (at 3.5-h intervals) might not have achieved CDS.

Two recent meta-analyses (Baker et al. 2009; Stowe et al. 2008) support the 2006 EFNS/MDS recommendations on dopamine agonists for prevention of motor complications. For oral IR dopamine-agonist treatment compared with levodopa, one study (Stowe et al. 2008) reported an odds ratio (OR) of 0.51 (95 % CI: 0.43, 0.59; *p* < 0.00001) for risk of dyskinesia and 0.75 (95 % CI: 0.63, 0.90; *p* = 0.002) for risk of motor fluctuations. In the other study (Baker et al. 2009),while the UPDRS motor scores demonstrated that patients receiving dopamine agonists had a significantly inferior response compared with patients receiving levodopa, based on a >4-point higher ADL score (weighted mean difference, 4.69; 95 % CI: 3.76, 5.61; *p* < 0.0001), the ORs for risk of dyskinesia and “wearing off” were 0.36 (95 % CI: 0.22, 0.60; *p* < 0.0001) and 0.52 (95 % CI: 0.40, 0.66; *p* < 0.0001), respectively. Both meta-analyses found that dopamine agonists conferred an increased risk for somnolence, dizziness, nausea, and hallucinations. In a recent double-blind study of adding ropinirole PR in levodopa-treated patients with early PD (Watts et al. 2010), onset of dyskinesia was significantly delayed in the ropinirole PR group compared with a group receiving additional levodopa (hazard ratio, 6.46; *p* < 0.001). In a head-to-head comparison between transdermal rotigotine and oral ropinirole IR in early PD (Giladi et al. 2007), the patch failed to demonstrate noninferiority on UPDRS motor plus ADL scores. Differences in rates of motor complications were not assessed.

### Treatment of motor complications in advanced PD

In a literature review published in 2006 (Pahwa et al. 2006), a subcommittee of the American Academy of Neurology (AAN) concluded that entacapone and rasagiline had established their efficacy, and that pramipexole, ropinirole, and tolcapone were “probably” effective, for reducing “OFF” time in PD patients with motor fluctuations. For “OFF” time reduction, levodopa/carbidopa CR was not considered to be more effective than the IR formulation. Continuous-infusion therapies (i.e., apomorphine or LCIG) were not included in the analyses.

More recent meta-analyses of treatments adjunctive to levodopa in advanced PD have confirmed the capacities of oral IR dopamine agonists, COMT inhibitors, and MAO-B inhibitors to reduce “OFF” time and UPDRS scores. For all three drug classes, the improvements were at the expense of an increase in dyskinesia, compared with placebo (Stowe et al. 2010; Talati et al. 2009). By indirect comparisons of the three classes, IR dopamine agonists appeared to be the most effective for reducing “OFF” time (at −1.54 h/day vs. −0.83 for COMT inhibitors and −0.93 for MAO-B inhibitors), but the agonists (and the COMT inhibitors) carried a higher risk for dyskinesias than did the MAO-B inhibitors (Stowe et al. 2010).

In recent studies with placebo control (Pahwa et al. 2007; Schapira et al. 2011), slow-release oral formulations of ropinirole and pramipexole, taken adjunctive to levodopa, have also shown efficacy for reducing “OFF” time and UPDRS scores. For both agents, “ON” time without troublesome dyskinesia was significantly increased. However, the incidence of dyskinesia as an adverse event was higher (at 13 % for ropinirole PR vs. 3 % for placebo; and at 17 % for pramipexole ER vs. 8 % for placebo, a difference not tested statistically in either trial). In a recent 24-week study comparing ropinirole formulations (Stocchi et al. 2011), the slow-release form had a response rate (defined by ≥20 % reduction in “OFF” time; adjusted OR, 1.82; 95 % CI: 1.16, 2.86; *p* = 0.009) and a capacity to reduce UPDRS motor scores (adjusted mean change from baseline for ropinirole PR vs IR, −10.2 vs. −7.9, respectively; *p* = 0.022) significantly greater than those for the IR form. The incidence of adverse events was numerically higher among recipients of the slow-release form (who also reached higher agonist dosage, but lower levodopa dosage, than in the IR arm). In an analogous comparison (Schapira et al. 2011), pramipexole ER showed capacities to reduce “OFF” time and UPDRS motor plus ADL scores resembling those of pramipexole IR. The incidence of adverse events was numerically lower for the ER form (at similar agonist dosage and lower levodopa dosage than in the IR arm).

In each of two studies with placebo control (LeWitt et al. 2007; Poewe et al. 2007), transdermal rotigotine, adjunctive to levodopa, has shown efficacy for motor complications in advanced PD, as evidenced by significant reductions in “OFF” time (*p* ≤ 0.0031) and increases in “ON” time without troublesome dyskinesia (*p* ≤ 0.0078). Incidence rates for dyskinesia as an adverse event were higher for rotigotine than for placebo (but were not tested statistically). In one of these studies (Poewe et al. 2007), a third treatment arm permitted head-to-head comparisons between the rotigotine patch and pramipexole IR. Improvements in “OFF” time, “ON” time without troublesome dyskinesias, and other outcomes showed no significant differences between these two treatments.

No double-blind studies have yet been reported for chronic treatment with apomorphine. However, in 2004 a review of 11 long-term, uncontrolled, open-label studies of subcutaneous apomorphine infusion (Deleu et al. 2004) reported a 60 % mean reduction in “OFF” time (range 50–80 % across studies) and also improvement in dyskinesia, which, however, required a mean of 12 months (range 0.5–50 months) to reach its maximum. Two further studies of subcutaneous apomorphine infusion have since been reported. In a prospective study (Katzenschlager et al. 2005), 12 patients with motor fluctuations and disabling dyskinesias received apomorphine infusion for 6 months. “OFF” time was reduced from baseline by 38 % (*p* < 0.05) and dyskinesia duration by 40 % (*p* < 0.01). In four of the 12 patients, oral medication could be discontinued, and within this apomorphine monotherapy group, there was a significantly greater decrease in “OFF” time and dyskinesia severity and duration than in the polytherapy group (for “OFF” time, 64 vs. 18 %, respectively, *p* < 0.05; for dyskinesia severity and duration expressed as centimeters on a visual analogue scale, 8.6 vs. 19.8, respectively, *p* < 0.05). Skin nodules were reported in 11 of 12 patients, including two patients with skin changes and inflammatory reactions requiring rotation of the infusion site. A retrospective analysis (García-Ruiz et al. 2008) of 82 patients who tolerated subcutaneous apomorphine infusion for at least 3 months (mean, 20 months) also identified significant decreases from baseline in “OFF” time (mean baseline vs. last follow-up visit, 6.64 vs. 1.36 h/waking day, *p* < 0.0001) and dyskinesia severity score (1.65 vs. 1.15, respectively, *p* < 0.0006). In this study, 68 % of patients reported treatment-related skin nodules.

For LCIG, findings of a double-blind, double-dummy trial comparing intrajejunal gel infusion and oral administration of levodopa-carbidopa in IR form have now been presented. For 12 weeks, PD patients selected for having motor complications underwent active LCIG infusion and took placebo IR capsules or took active IR capsules and underwent placebo gel infusion (Olanow et al. 2011). Among 66 study completers (93 % of the 71 randomized subjects), decrease in “OFF” time and increase in “ON” time without troublesome dyskinesia favored LCIG by means of −1.91 and +1.86 h/d, respectively, while “ON” time with troublesome dyskinesia showed no significant change (Olanow et al. 2012). Significant global, functional (ADL), and quality-of-life LCIG benefits were also identified (Kieburtz et al. 2012).

Several previous, open-label studies had already evaluated LCIG during chronic treatment lasting up to 7 years (Table 1) (Antonini et al. 2007, 2008, 2010b; Eggert et al. 2008; Isacson et al. 2008; Nilsson et al. 2001). Although variations in trial design preclude any summarization of the numerical findings, measures of “OFF” time improved in all six studies, with significance achieved in all five studies in which statistical testing was performed; dyskinesia measures also improved in all six studies, with statistical significance in four of the five studies with statistical testing. In addition, a randomized crossover trial (Nyholm et al. 2005) has compared 3 weeks of individually optimized conventional treatment with 3 weeks of nasoduodenal LCIG infusion. The conventional treatment was oral levodopa/carbidopa in optional combinations with oral dopamine agonists, COMT-inhibitors, MAO-B inhibitors, amantadine, or subcutaneous apomorphine (by injections or infusion). Motor tasks were videotaped every 30 min for 8 h on 2 days during the second and third week of each treatment for rating of motor function by neurologists blinded to treatment identity. (To blind the conventional therapies, a dummy nasogastric tube was emplaced.) Of 24 enrolled patients, 20 completed conventional therapy and 21 completed LCIG. The mean percentage of ratings falling within the predefined range for a clinically desirable “ON” state was significantly greater during LCIG than during conventional therapy (at 90.7 vs. 74.5 %, respectively, *p* < 0.01), and the mean percentage of ratings of “OFF” state was significantly lower (at 1.8 vs. 19.2 %, respectively, *p* < 0.01). For ratings of “ON” with moderate-to-severe dyskinesia, LCIG and conventional therapy showed no significant difference. Of the four patients who received subcutaneous apomorphine infusion as part of their conventional therapy, two were rated as being in an “ON” state all or nearly all of the time on both conventional treatment and LCIG, but the other two had substantial improvements on LCIG. In one patient, the proportion of “ON” ratings improved from 56 to 94 %, and in the other from 68 to 100 %.

**Table 1:** “OFF” time and dyskinesia outcomes in prospective studies of long-term LCIG therapy

Study	*N* enrolled/*n* completed	Maximum duration	Outcome versus conventional treatment
Nilsson et al. (2001)	9/6^a^	7 years	“OFF” time duration decreased at 3–8 months and at 4–7 years^b^. Dyskinesia duration decreased at 3–8 months and further decreased at 4–7 years
Antonini et al. (2007)	9/7	1 year	“OFF” time and disabling-dyskinesia duration significantly decreased
Antonini et al. (2008)	22/17	2 years	“OFF”-time duration and severity (by UPDRS part IV) significantly decreased
Eggert et al. (2008)	13/11	6 months^c^	“OFF” time and “ON” time with disabling dyskinesia significantly reduced as percent of patient’s day
Isacson et al. (2008)^d^	14/12	6 months	Proportion of patients with reduced “OFF” time significantly greater; proportion with reduced dyskinesia numerically greater
Antonini et al. (2010b)	15/4	3 years	“OFF” time duration and dyskinesia severity (by UPDRS part IV) significantly decreased

*LCIG* levodopa/carbidopa intestinal gel, *UPDRS* unified Parkinson’s disease rating scale

^a^Six patients had video scoring of motor state at both of two time points. Changes were not tested statistically

^b^Excludes one patient at the final time point, who was considered “OFF” at all video recordings

^c^Some patients continued for up to 12 months

^d^Extension of Nyholm et al. (2005) crossover trial

The most common adverse events leading to discontinuation of chronic LCIG treatment have been related to medication delivery, including problems with tubing displacement or occlusion, the pump system, and stoma infections (Antonini et al. 2007, 2008, 2010b; Eggert et al. 2008; Nilsson et al. 2001). To monitor long-term safety, close observation is warranted, as guided by recent reports of neuropathy associated with homocysteine elevation and at least functional vitamin B12 and/or B6 deficiency (Klostermann et al. 2012).

### Treatment of nonmotor symptoms

In general, published management recommendations for nonmotor PD symptoms have not attempted to differentiate between symptoms intrinsic to PD and those arising as complications of PD therapy. A contributory problem may be that in clinical studies of dopaminergic options, improvement of nonmotor PD symptoms, levodopa-related or not, has seldom been either a primary endpoint or a means for comparing treatments with potentially different abilities to produce CDD. In a double-blind, 12-week, parallel-group study of patients with early PD (Fung et al. 2009), oral LCE was superior to levodopa/carbidopa for improving quality of life, as rated by total score on the eight-item PDQ (PDQ-8) (Jenkinson et al. 1997b) and by subscores for the PDQ-8 nonmotor domains of depression, personal relationships, communication, and stigma. In a small single-dose, crossover study of oral levodopa/carbidopa CR versus IR (Kulisevsky et al. 2007), the seven PD patients with “wearing-off” showed significant mood improvement after IR treatment, while the seven nonfluctuating patients showed no mood improvement after receiving either drug. Plasma levodopa concentration correlated with anxiety level but not with mood.

Transdermal rotigotine has recently been assessed versus placebo in a large, double-blind trial (Trenkwalder et al. 2011a) in PD patients with unsatisfactory early morning motor-symptom control. On the PDSS-2 (Trenkwalder et al. 2011b), the mean 12-week change in total score (a coprimary outcome) was significantly improved in the rotigotine group compared with placebo from baseline to end of maintenance [least squares (LS) mean treatment difference, −4.26; 95 % CI: −6.08, −2.45; *p* < 0.0001]. Difficulty in falling asleep and feeling tired and sleepy in the morning were among the ten items showing significant improvement (among the instrument’s total of 15). Significant improvement versus placebo was also documented for mean change in total score on the NMSS (Martinez-Martin et al. 2009) from baseline to end of treatment (LS mean treatment difference, −6.65; 95 % CI: −11.99, −1.31; *p* = 0.015), with significant changes on the sleep/fatigue and the mood/cognition domains (LS mean treatment differences of −2.03; 95 % CI: −3.31, −0.75; *p* = 0.002 and −3.40; 95 % CI: −5.22, −1.58; *p* = 0.0003, respectively), and on the BDI-II depressive-symptomatology scale (Visser et al. 2006) from baseline to end of treatment (LS mean treatment difference, −2.01; 95 % CI: −3.55, −0.47; *p* = 0.011).

Improvement of nonmotor PD symptoms by subcutaneous apomorphine infusion has been a primary endpoint in two small prospective trials (Martinez-Martin et al. 2010; Reuter et al. 1999). In six patients with refractory nocturnal symptoms (Reuter et al. 1999), continuous overnight infusion (with placebo control in three of the subjects) was associated with decreases in awakenings, dystonia, pain, and nocturia. In an open-label trial (Martinez-Martin et al. 2010), 17 patients with advanced PD received subcutaneous apomorphine infusion and 17 received conventional therapy. At approximately 6 months, NMSS and PDQ-8 total scores showed significant improvement from baseline in the apomorphine group but did not change in the conventional-therapy group.

Improvement of nonmotor PD symptoms by intraduodenal LCIG infusion was the primary endpoint in a prospective, open-label trial conducted in 22 patients with daily motor fluctuations and dyskinesia refractory to optimized conventional therapy with oral medications, transdermal rotigotine, or subcutaneous apomorphine infusion (Honig et al. 2009). After discontinuation of the conventional therapy (except for nighttime oral dosing with levodopa CR or a long-acting dopamine agonist) and its replacement by LCIG for a mean of 6.7 months, mean change in NMSS total score showed significant improvement from baseline (−50.5, *p* = 0.0001). Of the nine NMSS domains, six were significantly improved [cardiovascular (−2.41, *p* = 0.0004), sleep/fatigue (−11.32, *p* = 0.0001), attention/memory (−3.27, *p* = 0.002), gastrointestinal (−6.23, *p* = 0.0003), urinary (−6.64, *p* = 0.002), and miscellaneous (−7.73, *p* = 0.0004)]. The change in total NMSS score was correlated with changes on measures of motor function [UPDRS motor-complication score (−5.91, *p* = 0.0000), UPDRS dyskinesia subscore (−3.7, *p* = 0.0001), and “OFF” time as a proportion of the waking day (*r* = 0.54, *p* < 0.01)]. On the PDSS and PDQ-8, mean improvement in total score was also significant (+28.51; *p* = 0.002 and −23.4; *p* = 0.0003, respectively).

## Conclusions

**Fig. 6:**
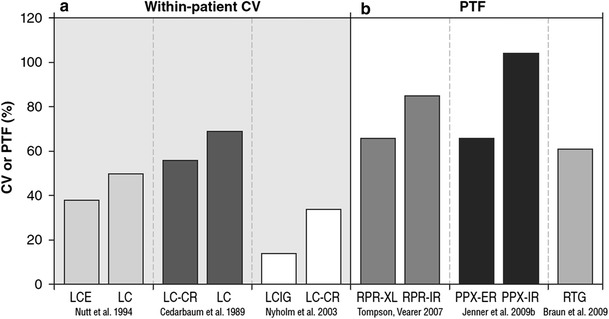
Variability in the plasma level of various formulations of dopaminergic therapies. Coefficient of variation (CV) (**a**)—the standard deviation in plasma level, expressed as a percentage of group geometric mean plasma level—for oral immediate-release levodopa/carbidopa/entacapone versus levodopa/carbidopa (LCE vs. LC), oral slow- versus immediate-release levodopa/carbidopa (LC-CR vs. LC), and intraduodenal levodopa/carbidopa intestinal gel versus oral slow-release levodopa/carbidopa (LCIG vs. LC-CR). Peak-to-trough fluctuation (PTF) (**b**)—*C*_max_ − *C*_min_, expressed as a percentage of group geometric mean plasma level—for oral slow- versus immediate-release ropinirole (RPR-XL vs. RPR-IR), oral slow- versus immediate-release pramipexole (PPX-ER vs. PPX-IR), and transdermal rotigotine (RTG). *CR* controlled-release, *ER and XL* extended-release

For several decades, the pharmacologic treatment of PD has been evolving, most recently in a quest to achieve CDS or, more verifiably, CDD. Improvements in the steadiness of the plasma concentration-versus-time profiles of various dopaminergic therapies (Fig. 6) may be a signal of progress. However, improvements in plasma profile do not necessarily translate into a more continuous stimulation of central dopamine receptors. To directly evaluate the degree of CDS that a dopaminergic drug may confer, studies assessing dopamine-receptor occupancy (as measured, e.g. by brain imaging) will be necessary (Brooks et al. 2010).

So far, clinical studies of efforts to improve PD motor symptoms using therapies expected to provide more CDS have generally been positive, including double-blind, randomized trials with placebo control. However, the findings of studies comparing differing active treatments have often failed to find evidence favoring these approaches over an intermittent therapy. In such trials, levodopa/carbidopa CR, for example, was not superior to levodopa/carbidopa IR in advanced PD (Pahwa et al. 2006); LCE was not superior to levodopa/carbidopa, at least for delaying motor complications in early PD (Stocchi et al. 2010); pramipexole ER was not superior to pramipexole IR as an adjunct to levodopa in advanced PD (Schapira et al. 2011); and transdermal rotigotine was not superior either to IR ropinirole as monotherapy in early PD (Giladi et al. 2007) or to IR pramipexole as an adjunct to levodopa in advanced PD (Poewe et al. 2007). A conceivable explanation for the lack of substantial difference may be that the so-called standard-release dopamine agonists in fact have “longish” half-lives compared with levodopa IR and even with levodopa CR. By a similar argument, continuous agonist delivery using transdermal rotigotine would lack superiority to IR ropinirole because the half-life of the oral IR agent is already fairly long. However, transdermal rotigotine is not as effective as the infusional dopaminergic therapies (apomorphine or levodopa), suggesting that the inherent potency of each agent is also determinative. In studies of differing formulations of the same agent, i.e., levodopa, the longer-acting formulation has shown superior benefit (Hauser 2012). For their part, the infusional therapies presumably gain from accessing the blood stream without need for gastric transit. Currently, apomorphine is the only dopamine agonist rivaling levodopa in potency, and its half-life is shorter. In the MPTP primate PD model, its intermittent administration shares with levodopa a capacity to induce dyskinesia, but in the same model, animals undergoing continuous subcutaneous apomorphine infusion for up to 6 months did not become dyskinetic (Bibbiani et al. 2005). In PD patients, moreover, continuous apomorphine infusion has been found to downregulate preexisting dyskinesia (Colzi et al. 1998; Katzenschlager et al. 2005).

Clinical studies focusing on improving nonmotor PD symptoms are becoming more common. These trials, too, have not yet clarified any potential differences across therapies with differing capacities for CDD. Nevertheless, the findings of nonmotor improvement among recipients of subcutaneous apomorphine (Martinez-Martin et al. 2010) or intrajejunal LCIG (Honig et al. 2009) suggest that nonmotor PD symptoms or complications may improve in tandem with the expected motor improvements. For more persistent nonmotor problems, nondopaminergic treatments seem likely to remain key (Zesiewicz et al. 2010). Here, too, future research should explore drug activity at dopaminergic synapses, so as to determine whether CDS is, in fact, an important determinant of clinical efficacy. Certainly, the complexities of optimal PD management, and the rationale for an underlying strategy such as CDS or CDD, have not yet been thoroughly elucidated.
